# Editorial: Environmental Catalysis and the Corresponding Catalytic Mechanism

**DOI:** 10.3389/fchem.2019.00075

**Published:** 2019-02-13

**Authors:** Zhimin Ao, Hongqi Sun, Andres Fullana

**Affiliations:** ^1^Guangzhou Key Laboratory of Environmental Catalysis and Pollution Control, Guangdong Key Laboratory of Environmental Catalysis and Health Risk Control, School of Environmental Science and Engineering, Institute of Environmental Health and Pollution Control, Guangdong University of Technology, Guangzhou, China; ^2^School of Engineering, Edith Cowan University, Joondalup, WA, Australia; ^3^Department of Chemical Engineering, University of Alicante, Alicante, Spain

**Keywords:** environment remediation, catalysts, clean energy, photocatalysis, biomass conversion, density functional theory, catalytic mechanism

The ever growing environmental pollution has stimulated the rapid development of environmental catalysis in recent years. Environmental catalysis is a multidisciplinary research field for which more and more chemists, materials scientists, as well as environmentalists have devoted their efforts working in this field because of the bright potentials in improving human health and life quality. With the progresses in controllable materials synthesis, advanced characterizations (electron microscopy, spectroscopy, etc.), high-level analytical chemistry, together with the computational studies, catalysis continues to be the driving force for generation of clean energy, abatement of major pollutants in air and water, and meantime the theories behind the catalytic reactions are illustrated. In the current Research Topic, an elegant collection of original research and review articles reporting the synthesis of high-performance catalysts, their applications in various catalytic technologies for environment remediation, and relevant theoretical calculations for understanding the catalytic mechanisms is presented.

Photocatalysis remains to be a research focus for the environmental catalysis community due to the wide applications in carbon dioxide reduction, oxidation of volatile organic compounds (VOCs), elimination of aqueous organic pollutants and disinfection, water splitting (Chen et al., 2011; Zheng et al., 2016; Wang et al., 2017). Particularly, the synthesis of visible light and near-infrared (NIR) light responsive semiconductor photocatalysts is of immense interests to scientists because visible light and NIR light occupy around 90% of the solar light energy, in comparison to the no more than 5% for ultraviolet light (UV). To enhance the visible light absorption of the most popularly used TiO_2_, several routes including creating defects and element doping are developed Qin and Lin et al., introduced carbon and nitrogen elementals into TiO_2_ simultaneously and demonstrated the improved catalytic property of such C, N-TiO_2_ catalyst compared to anatase TiO_2_ under simulated sunlight irradiation for degradation of 4-nitrophenol. Moreover, they also evaluated the embryonic toxicity of intermediate degradation compounds (Osin et al.). Silva et al., synthesized Pd-Cu loaded over hybrid materials of carbon nanotubes and TiO_2_ for nitrate reduction. It reported that the Pd-Cu loaded on the hybrid materials have high photocatalytic performance for ${\text{NO}}_{3}^{-}$ conversion (Silva et al.). Other than TiO_2_, the bismuth compounds as a new class of photocatalytic materials have been paid much attention in recently. Among them, bismuth oxychloxide (BiOCl) exhibits excellent photocatalysis behaviors driven by UV light. To expand its light absorption to visible light spectrum, efforts to fabricate graphene oxide/BiOCl nanocomposite film and BiOI/BiOCl film are reported by Zhu and Zhang et al., respectively (Lin et al.; Zhong et al.). BiVO_4_ is found to be a visible light-activated photocatalyst due to the narrower band gap than TiO_2_, the involvement of graphene oxide or BiOI can further improve the photocatalytic performance for RhB degradation. However, the poor specific surface area limits its catalytic performance. To this end, Channei et al. utilized a co-precipitation method to coat SiO_2_ onto BiVO_4_ and obtained SiO_2_/BiVO_4_ composites with larger surface area and higher photocatalytic activity and degradation efficiency towards methylene blue dye with respect to monoclinic BiVO_4_ (Channei et al.). Furthermore, a comprehensive overview on semiconductors loaded with carbon cocatalysts as photocatalysts for water splitting and pollutant degradation was provided by Peng et al., aiming to give a clue to the rational design of low-cost photocatalysts with more efficient solar light utilization. The synthesis methods of various types of carbon-semiconductor composite photocatalysts were summarized, the contribution of different carbon allotropes like C_60_, carbon nanotubes, graphene to the enhanced photocatalytic activity were compared and the cocatalytic effect mechanisms were discussed (Han et al.).

Catalytic conversion of biomass to biofuels is another emerging topic in the environmental catalysis field out of the urge to transform the agricultural wastes into resources and to reduce the carbon dioxide emission from fossil fuels combustion (Wang and Xiao, 2015; Xiong et al., 2015). Heterogeneous acidic catalysts and enzyme biocatalysts are among the leading candidates for the conversion of lignocellulosic biomass to fuels and value-added chemicals. One relevant paper presented the good catalytic performance of bifunctional catalysts which were synthesized by decorating propyl/phenyl-sulfonic acid group functionalized mesoporous silica materials SBA-15 with Pt particles in the reaction of hydrodeoxygenation of bio-derived phenol to produce cyclohexane fuel (Mo et al.).

Pharmaceutical and personal care products (PPCPs) are emerging contaminants, which are widely present in pharmaceutical and hospital wastewater, even natural water. In this Research Topic, Fe-MCM-41s were fabricated at different conditions to adsorb widely used antibiotics ciprofloxacin hydrochloride (CPX) for its removal from waste water (Wu et al.). Owing to the rise of supercomputers, computational studies become important complementary tools for elucidation of the inherent mechanisms of catalytic reactions. Currently *Ab initio* techniques like Density Functional Theory (DFT) are popularly adopted for theoretical calculations. Here in this Research Topic, two examples of DFT studies on the mechanisms of CO oxidation catalyzed by Mn-embedded divacancy graphene and boosted oxygen reduction reaction performance catalyzed by two-dimensional metal–organic frameworks TM_3_(hexaiminotriphenylene)_2_ monolayer (where TM = Ni, Co, Fe, Pd, Rh, Ru, Pt, Ir, and Os) were shown (Jiang et al.; Xiao et al.).

We believe that this collection, Environmental Catalysis and the Corresponding Catalytic Mechanism, illustrates the advancements of catalysis for environment remediation and innovations in clean energy with diminished production of undesired by-products. At the same time, challenges and perspectives for this field are also addressed with the hope that future interests will be focused to help establish a world with clean air and water, as well as sustainable and green energy for human to live in.

## Author Contributions

ZA drafted the manuscript. HS and AF made substantial improvements, and all authors approved for publication.

### Conflict of Interest Statement

The authors declare that the research was conducted in the absence of any commercial or financial relationships that could be construed as a potential conflict of interest.

## References

[B1] Chen X. Liu L. Yu P. Y. Mao S. S. (2011). Increasing solar absorption for photocatalysis with black hydrogenated titanium dioxide nanocrystals. Science 331, 746–750. 10.1126/science.1200448 21252313

[B2] Wang L. Xiao F.-S. (2015). Nanoporous catalysts for biomass conversion. Green Chem. 17, 24–39. 10.1039/C4GC01622J

[B3] Wang X. Wang F. Sang Y. Liu H. (2017). Full-Spectrum Solar-Light-Activated Photocatalysts for light–chemical energy conversion. Adv. Energy Mat. 7:1700473 10.1002/aenm.201700473

[B4] Xiong H. Schwartz T. J. Andersen N. I. Dumesic J. A. Datye A. K. (2015). Graphitic-carbon layers on oxides: toward stable heterogeneous catalysts for biomass conversion reactions. Angew. Chem. Int. Edition 54, 7939–7943. 10.1002/anie.201502206 25973732

[B5] Zheng Q. Durkin D. P. Elenewski J. E. Sun Y. Banek N. A. Hua L.. (2016). Visible-light-responsive graphitic carbon nitride: rational design and photocatalytic applications for water treatment. Environ. Sci. Technol. 50, 12938–12948. 10.1021/acs.est.6b02579 27934277

